# Chemokine RANTES/CCL5 as an unknown link between wound healing in the jawbone and systemic disease: is prediction and tailored treatments in the horizon?

**DOI:** 10.1186/s13167-015-0032-4

**Published:** 2015-05-06

**Authors:** Johann Lechner, Volker von Baehr

**Affiliations:** Clinic for Integrative Dentistry, Gruenwalder Str. 10A, 81547 Munich, Germany; Compartment of Immunology and Allergology, Institute for Medical Diagnostics, MVZ GbR, Nicolaistrasse 22, 12247 Berlin, Germany

**Keywords:** Chronic inflammation in the jawbone, Fatty necrotic osteolytic jawbone, Hyperactivated signaling pathways, RANTES/CCL5, Predictive preventive personalized medicine

## Abstract

**Background:**

This research elucidates the question of whether common and widespread dental procedures (DP) like root filling (RF) and the removal of wisdom teeth (WT) contribute to chronic inflammation in the jawbone. Dentists, in carrying out these DP, can set off defective wound healing in the jawbone in ignorance of its connection to inflammatory mediators and the possibility of it being a hidden cause of chronic systemic diseases (SYD).

**Materials and methods:**

We examined samples of the jawbone for seven cytokines by multiplex analysis in three groups of jawbone areas. In order to clarify systemic interrelations, specimens from 16 patients were analyzed in areas of former surgery in the retromolar wisdom tooth area; specimens from 16 patients were analyzed in the jawbone, apically of teeth with RF; and specimens from 19 patients were of the healthy jawbone. Each of the retromolar and the apical jawbone samples showed clinically fatty degenerated and osteonecrotic medullary changes.

**Results:**

All fatty necrotic and osteolytic jawbone (FDOJ) samples showed regulated on activation, normal T-cell expressed and secreted (RANTES) and fibroblast growth factor (FGF)-2 as the only extremely overexpressed cytokines. FDOJ cohorts showed a 30-fold mean overexpression of RANTES and a 20-fold overexpressed level of FGF-2 when compared to healthy controls.

**Conclusions:**

As RANTES is discussed in the literature as a possible contributor to inflammatory diseases, and though it might have oncogenic effects, we hypothesize that FDOJ in areas of improper and incomplete wound healing in the jawbone might act as hyperactivated signaling pathways, while serving as an unknown source of “silent inflammation”. Because of the wide range of RANTES in immune diseases, treating FDOJ can cover many potential prediction or prognosis of individual outcomes.

## Overview

Acute disease is unavoidable given our interaction with dental decay, but it is just the tip of a disease iceberg. Below the surface lie hidden chronic diseases (cancer, autoimmune diseases, etc.), the products of an immune system that is being constantly triggered by overexpressed cytokines. These triggers lead to the stimulation of different signaling pathways, which are instrumental in the development of chronic disease. In general, the cell communication systems are organized as cascades in sequential stages [1]. The signal messengers, like the cytokines, carry instructions that are received by cells with specific receptors, which are able to detect them. Most dental procedures consist in eliminating acute inflammation in situations that do not feature typical signs of inflammation like pain and tissue swelling. This is the case with root fillings (RF) and surgical procedures like wisdom tooth surgery (WTS). The use of antibiotics helps the dentist and the patient overcome inflammation after dental procedures and during acute infections (AIs) in daily practice. This research tries to elucidate the conversion of AI into chronic inflammation (CI) in the jawbone during common dental procedures like RF and WTS. In daily dental practice, the effects of CI on overall health are normally not of interest because local problems seem to be resolved after the symptoms of AI are gone. Here, we try to define the characteristics of CI by reference to the possible cytokine content of fatty degenerative osteonecrotic jawbone (FDOJ) found in old WTS extraction or RF operation sites with insufficient wound healing (IWH). Our hypothesis is that the reduction of AI might serve as the beginning of a possible development of CI in the jawbone. Persons with certain risk factors might be prone to developing subsequent systemic diseases (SYD). Treatments tailored to the person and individually targeted prevention is a crucial part of this therapeutic concept.

## Methods

### Groups of patients examined

In this study, we focused on IWH in former wisdom tooth areas that were mostly in danger of developing AI following dental surgery in a group of patients with rheumatoid arthritis (RA) (number [*n*] = 16). The inclusion criteria for the studied population with RA were (1) patients with clinical symptoms of joint pain, (2) a diagnosis of “rheumatoid arthritis” as determined by internal doctors, and (3) a local diagnosis of FDOJ in the jawbone in retromolar areas with former WTS. Demographic data of the RA/WTS cohort were as follows: an average age of 56 years (standard deviation [SD] = 11.4 years) and a gender ratio of 8:8 (females/males). We also focused on areas of the jawbone underneath RF in a group of patients with the following SYD (*n* = 16): RA = 6; neurodegenerative diseases like chronic fatigue syndrome and amyotrophic lateral sclerosis = 4; allergies = 2; breast cancer (BC) = 2; and Hashimoto’s thyroiditis = 2. The inclusion criteria of the studied cohort with root-filled tooth (RFT) included patients with systemic immunological or neurodegenerative diseases and a local diagnosis of FDOJ in the jawbone, apically, of one RF. Demographic data of the RF/SYD cohort included an average age of 60 years (SD = 13.2 years) and a gender ratio of 14:1 (females/males).

We collected tissue samples from these 32 patients (RA/WTS group = 16; RF/SYD group = 16) from FDOJ regions. Mandatory inclusion criteria for both groups were the availability of two-dimensional orthopantomograms (2D-OPG), cone beam 3D-DVT, and measurement of the bone density of the jawbone with transalveolar ultrasound (TAU) technology. TAU is a useful tool when establishing FDOJ [2].

The third cohort was a group of patients with samples of the healthy jawbone (HJB), which were taken in the form of drill cores during normal dental implantation surgery. Inclusion criteria for this group were no radiologically distinctive feature in 2D-OPG and inconspicuous TAU measurements of bone density in the implantation range. The age range of the control group consisting of 19 patients without FDOJ was 38–71 years, with an average age of 54 years (SD = 12.4 years), and there was a gender ratio (female/male) of 11/8.

The taking by patients of any medications due to systemic complaints did not serve as an exclusion criterion. Use of bisphosphonate medication was the central exclusion criterion for all three groups. The research was based on data retrieved from patients during normal dental surgery. All patients gave their written informed consent. This study was performed as a randomized controlled trial. Statistical analyses were performed using IBM SPSS, version 19 (IBM Corporation, Armonk, NY, USA).

### Clinical features of fatty degenerative jawbone definition and diagnostic criteria

**Figure 1:**
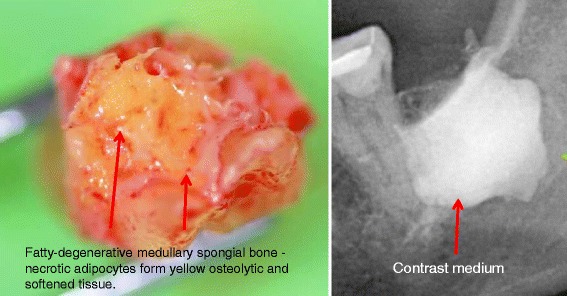
FDOJ sample of fatty and osteolytic degenerated bone marrow (left part) and a contrast medium in the FDOJ cavity after curettage (right part).

The softening in FDOJ bone marrow is so distinct that the marrow space can actually be sucked and spooned out. Hollow cavitations with fatty degenerated adipocytes have undergone dystrophic changes, accompanied by demyelination of the bony sheath of the infra-alveolar nerve. All 32 FDOJ samples in the wisdom tooth (WT) and RF groups presented themselves clinically and macroscopically as fatty lumps. Figure 1 shows a type of specimen with a predominantly fatty transformation of the jawbone in the left part. The often impressive extent of FDOJ lesions is documented in the right part by X-ray with a contrast medium.

**Figure 2:**
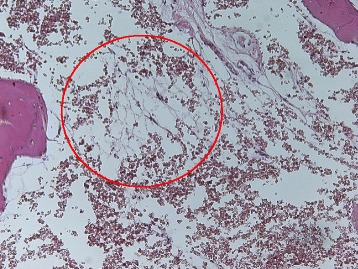
Photomicrograph of an FDOJ lesion with typical signs of osteonecrosis and fatty degenerated and necrobiotic adipocytes, centered in a hollow cavitation of the jawbone. The red circle shows fatty degenerated and necrobiotic adipocytes centered in a hollow cavitation of the jawbone with clear signs of osteonecrosis (1:200).

To obtain a better understanding of this marrow disorder, Figure 2 shows a characteristic photomicrograph of FDOJ lesions. Following previous research [3], it can be seen that FDOJ is a similar lesion to those found in the long bones, which are primarily defined by bone marrow edema and chronic nonsuppurative osteomyelitis.

### Sampling of FDOJ tissue

Current treatment of the FDOJ lesion consists of curettage of the bony cavity [4,5]. To elucidate the cytokine patterns in the jawbone in the corresponding author’s Clinic for Integrative Dentistry, 32 patients diagnosed with FDOJ in sites of former WTS or in the apical area of RFT had surgery on the affected area of the jaw. Following local anesthesia and the folding of a mucoperiostal flap, the cortical layer was removed. All patients showed FDOJ inside the bone marrow, which was quite similar to the samples described in literature [6,7] and as illustrated in Figure 1. In all cases, surgery was performed on edentulous jaw areas in the regions of former WT, adjacent retromolar areas, or the area underneath teeth with root fillings. The FDOJ samples with a volume up to 0.5 cm^3^ were stored in a dry, sterile, 2-mL collecting vial (Sarstedt AG & Co., Nümbrecht, Germany), which was made airtight, and frozen at −20°C.

### Processing of necrotic tissue samples and measurement of cytokines

At the examining Institute for Medical Diagnostics, Nikolaistr. 22, D-12247 Berlin (inspected by DAKKS [Deutsche Akkreditierungsstelle GmbH; accredited to DIN EN ISO/IEC 17025:2005 and DIN EN ISO 15189:2007]), the samples were homogenized by mechanical force in 200 μL of cold protease inhibitor buffer (Complete Mini Protease Inhibitor Cocktail; Roche Diagnostics GmbH, Penzberg, Germany). The homogenate was centrifuged for 15 min at 13,400 rpm. Afterwards, the supernatant was collected and centrifuged for a further 25 min at 13,400 rpm. In the 15 supernatants of tissue homogenate, we measured regulated on activation, normal T-cell expressed and secreted (RANTES), fibroblast growth factor (FGF)-2, interleukin (IL)-1 receptor antagonist (ra), IL-6, IL-8, monocyte chemotactic protein-1 (MCP1), and tumor necrosis factor-alpha (TNF-α). Measurement was performed using the Human Cytokine/Chemokine Panel I (MPXHCYTO-60K; Merck KGaA, Darmstadt, Germany) according to the manufacturer’s instructions, and these findings were analyzed using the Luminex® 200™ with xPonent® Software (Luminex Co, Austin, TX, USA).

### Results of seven cytokine panel evaluations in the osteonecrotic and healthy jawbone

**Figure 3:**
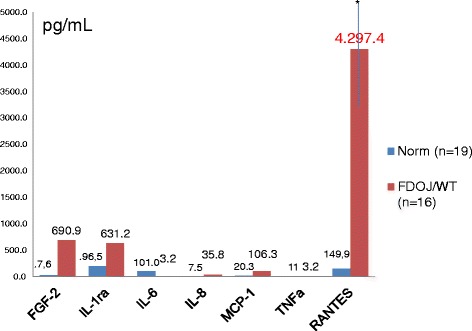
Distribution of seven cytokines in HJB (*n* = 19) (pg/mL) and in FDOJ (*n* = 16).

The mean values of 19 samples of HJB were as follows (pg/mL): FGF-2, 27.6; IL-1ra, 196.5; IL-6, 101.0; IL-8, 7.5; MCP-1, 20.3; TNF-α, 11.0; RANTES, 149.9 (see Figure 3). Values for healthy patients and HJB were not available for comparison from the literature.

### Expression of seven cytokines in the wisdom tooth area of the jawbone in 16 patients with rheumatoid arthritis

The results of the multiplex analysis of the seven cytokines in the WTS/RA cohort (*n* = 16) are shown in Figure 3, and they were compared to the mean values of HJB. WTS/RA patients show elevated inflammatory signals in the FDOJ samples deriving from the WTS areas with obvious IWH in the jawbone with an average RANTES/CCL5 value of 4.297,4 pg/mL (SD = ±2,145.7) when compared to that of the randomized controlled sample of 149.9 pg/mL in HJB. All other cytokines—except FGF-2 and IL-1ra—were not derailed. The most striking result of this analysis is the high concentration of RANTES. No other proinflammatory messengers, such as IL-6, IL-8, MCP-1, or TNF-α, were detected at such elevated levels from the jawbones of a group of patients with former WTS and RA.

### Case report: RANTES overexpression in the jawbone in the former wisdom tooth area

**Figure 4:**
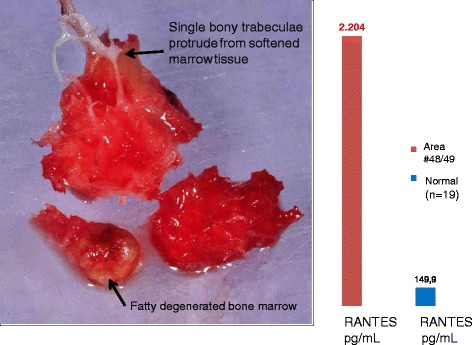
FDOJ sample from the former area of WTS with focal loss of medullary bone structure, as well as fatty changes in the remaining osteoporotic marrow defect. Single bony trabeculae protrude from the softened and yellowish altered surrounding marrow tissue.

The FDOJ sample in Figure 4 was removed from a 36-year-old patient; her right shoulder had been treated with cortisone injections because of RA. The picture shows the FDOJ sample after surgery in the right lower wisdom tooth area, with focal loss of the medullary bone structure, with ischemic and fatty changes in the remaining osteoporotic marrow defect. Single bony trabeculae protrude from the softened and yellowish altered surrounding marrow tissue. In the columns in the right image, multiplex analysis of this FDOJ sample indicated a 15-fold overexpression of RANTES in the retromolar area with former WTS when compared to HJB. Inflammatory pain in the patient’s right shoulder disappeared shortly after curetting this FDOJ.

### Expression of seven cytokines in the apical area of root-filled teeth in the jawbone

**Figure 5:**
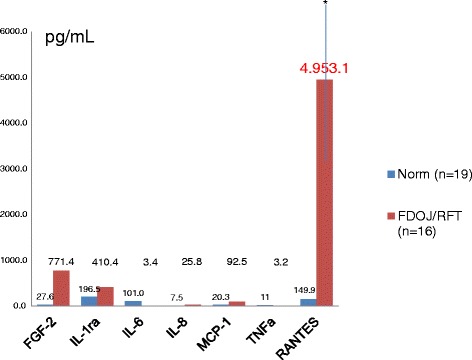
Distribution of seven cytokines in HJB (*n* = 19) (pg/mL) and in FDOJ (*n* = 16) from the jawbones of a group of patients with SYD and RFT.

The results of the multiplex analysis of the seven cytokines in the RFT/SYD cohort (*n* = 16) are shown in Figure 5 and compared to mean values of HJB. RFT/SYD patients show elevated inflammatory signals in the FDOJ samples that are derived from the RFT areas, and obvious IWH is evident in the jawbone with an average RANTES/CCL5 value of 4,953.1 pg/mL (SD = ±2,645.2) when compared to that of the randomized controlled sample of 149.9 pg/mL in HJB. All other cytokines—except FGF-2 and IL-1ra—were not derailed. The most striking result of this analysis is the high concentration of RANTES. No other proinflammatory messengers, such as IL-6, IL-8, MCP-1, or TNF-α, were detected at such elevated levels.

### Case report: RANTES overexpression in the jawbone underneath a root-filled tooth

**Figure 6:**
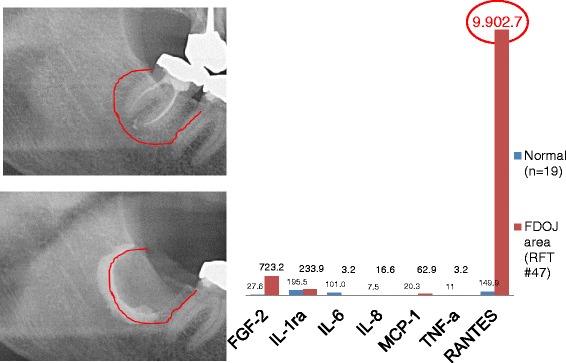
Comparison of seven cytokines in FDOJ underneath RFT #47 with the cytokines in the healthy jawbone (*n* = 19). Intraoperative documentation of extension of FDOJ in the right lower jawbone, area #47 apically of RFT #47, by contrast agent after the surgical removal of RFT #47.

In order to document the noncoincidental representation of FDOJ in normal dental 2D-OPG and to determine the intramedullary extent of FDOJ, we injected a contrast agent into the FDOJ area after curetting the softened cancellous bone underneath the previously extracted RFT (see Figure 6). The upper X-ray shows the same area with inconspicuous bony structures; optical analysis of the X-ray diagnosed a “normal/healthy” jawbone. The lower X-ray in Figure 6 shows the dimension of the contrast agent filling up the FDOJ after curetting of FDOJ, which was not adequately recorded by the X-ray before surgery. The right columns compare RANTES expression for this FDOJ area (RFT #47) with RANTES in HJB (*n* = 19).

### Case report: recurring breast cancer and comparison of RANTES overexpression in the wisdom tooth area and in the jawbone underneath a root-filled tooth

**Figure 7:**
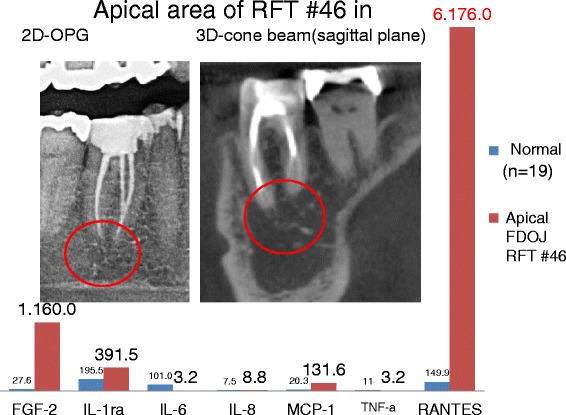
Comparison of the seven cytokines in the FDOJ underneath RFT #46 with the cytokines in the healthy jawbone (*n* = 19). The left X-ray shows the apical area of RFT #46 in a red circle on a two-dimensional orthopantomogram (2D-OPG); the right X-ray shows the same area on the sagittal plane in three-dimensional cone beam (3D-CB).

**Figure 8:**
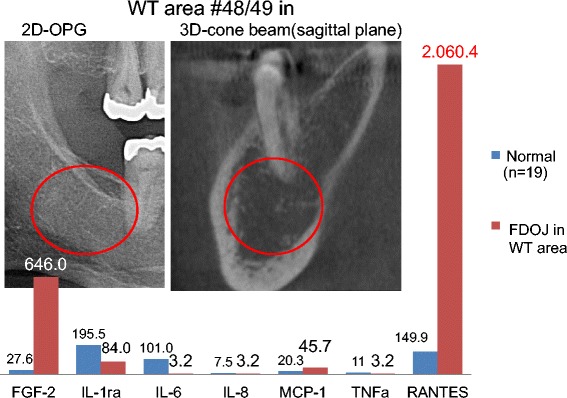
Comparison of seven cytokines in the FDOJ in area #48/49 with cytokines in a healthy jawbone (*n* = 19). The left X-ray shows the retromolar WT area on a two-dimensional orthopantomogram (2D-OPG); the right X-ray shows the same area in the sagittal plane in three-dimensional cone beam (3D-CB).

The patient, 69 years old, had recurring BC on the right side. This case is of interest because of the difference in RANTES expression in the jawbone underneath endodontically treated tooth #46 (US #30) and in retromolar area #48/49 (US #32). In Figure 7, X-rays show the inconspicuous apical area of RFT #46 with only very discrete changes in the trabecular structures. The columns in the same figure compare the distribution of seven cytokines in this FDOJ sample (in red) and 19 healthy controls (in blue). The X-rays in Figure 8 show the inconspicuous retromolar wisdom tooth area #48/49. As in Figure 7, the columns in Figure 8 compare the distribution of seven cytokines in the FDOJ sample of wisdom tooth area #48/49 (in red) and in 19 healthy controls (in blue). Amazingly, RANTES expression in area #46 underneath the RFT was threefold higher (6,178 pg/mL) than RANTES expression in area #48/49 (only 2,060 pg/mL), with obvious IWH in the former area of WTS. Please note again the comprehensive failing of any X-ray diagnosis in both of the presented jawbone areas.

## Results

### Role of RANTES in inflammatory disease

RANTES belongs to the family of chemotactic cytokines known as CC pattern chemokines. It is expressed by an early response gene. RANTES is chemotactic for T-cells, eosinophils, and basophils, and it plays a key role in recruiting leukocytes to inflammatory sites. The significance of RANTES for the development of diseases seems to be enormous; RANTES interferes with immune responses on a number of levels and therefore plays a crucial role in pathological states. The chemotactic properties of RANTES send T-cells, dendritic cells, eosinophils, natural killer (NK) cells, mast cells, and basophils to the sites of inflammation and infection [8]. RANTES is also an effective activator of leukocytes, which play a key role in a wide range of inflammatory disorders [9] (i.e., in RA) [10]. The role of RANTES as a key mediator in RA is well known in medical science; RANTES is secreted by human fibroblasts in the synovia and therefore can be part of a progressive inflammatory process that accompanies RA [11]. RA is characterized by the recruitment of leukocytes from the vasculature into inflamed synovial tissue and synovial fluid, which depends, in part, upon the continued maintenance of chemotactic stimuli. The selective chemoattractant and activation effects of chemokines on leucocytes identify them as potentially ideal candidates for mediating selective inflammatory processes in RA [12].

In their studies, other researchers have demonstrated that by expressing RANTES and IL-8, synovial fibroblasts may participate in the ongoing inflammatory process in RA. In addition, the observation that the gene coding for these chemokines is differentially regulated, depending upon the presence of different cytokines, indicates that the type of cellular infiltrate and the progress of the inflammatory disease is likely to depend upon the relative levels of stimulatory and inhibitory cytokines [10]. Synoviocytes produce synovial fluid and secrete many of these effector molecules, which advance inflammation and joint destruction [13]. They form part of a complex network of autocrine and paracrine factors. Wang et al. reported that there is increasing evidence suggesting a role for RANTES in the pathogenesis of RA, and they further evaluated the possible effect of the RANTES gene on the susceptibility to RA in Chinese patients. Their research indicated that a polymorphism in the promoter region of the RANTES gene is associated with susceptibility to RA in the Chinese population [14]. This conclusion may explain the fact that while many people have FDOJ lesions in their jawbone, only a certain percentage suffer from RA. Differences in genetically defined susceptibility to RANTES seem to be one factor that determines which disease—out of many connected to high RANTES levels—might be induced by signaling from FDOJ-derived RANTES.

A summary of the results of these studies provides evidence that RANTES is a key mediator in all joint inflammation. RANTES has also been associated with the induction or promotion of cancer [15]. RANTES levels were markedly elevated in the primary tumor and metastatic lesions of all patients with breast and cervical cancer. The high incidence and intensity of RANTES expression has been noted in advanced BC. RANTES expression was analyzed in parallel to disease progression, which was positive upon diagnosis and was predictive of clinical course. These results suggest that the assessment of RANTES expression may be a useful prognostic indicator for the identification of patients with an apparently poor prognosis [16]. The development of BC may, in part, be due to the ability of RANTES to act directly on the tumor cells and to promote tumor progression [17].

### Origin of RANTES in FDOJ and adipocytes

That the inflammatory response of adipose tissue is associated with a systemic inflammatory response is well known and widely discussed. In obesity, this systemic inflammatory response originates in the adipose tissue itself. Secretion of inflammatory cytokines mediates the systemic effects of adipose tissue inflammation. Huber et al. found an increased expression of RANTES in fatty tissue in obese patients [18]. Our findings regarding RANTES/CCL5 secretion by FDOJ deserve further discussion: reduced blood flow and capillary density followed by ischemia in the jawbone may lead to a hypoxic situation [19]. Adipocytes and the necrotic parts of fat cells are considered by many studies to be immunologically effective ingredients. The role of these immune effects on understanding FDOJ, RANTES/CCL5, and SYD is an evident issue and needs further illumination in the discussion. While proinflammatory cytokines such as TNF-α, IL-6, and prostaglandins are already distributed early in the acute stage of an injury or tissue infection, there are many indications that chemokines like RANTES are activated at a later time and that they can act in the conversion of acute pain into a more chronic phenomenon. In fact, recent data suggest that, in conjunction with tissue damage or infection, ischemia-induced chemokine expression causes an increase in inflammatory cytokines [20].

### FDOJ as a systemic threat from RANTES overexpression

FDOJ is similar to silent inflammation or subclinical inflammation without typical signs of acute inflammation. In CI, the local production of proinflammatory cytokines overstrains regulatory and compensating mechanisms by building FDOJ in the bone marrow. This phenomenon seems to be more widespread than dentists and doctors presumed in the past. It is generally accepted that an imbalance between cytokines and their specific inhibitors is characteristic of chronic inflammatory conditions [21]. Cytokines merge to release the immune response and to induce acute inflammatory events in the transition or persistence of the CI. This means that when maintaining healthy conditions, the cytokine-producing mechanisms must be controlled [22]. FDOJ represents a new cellular response phenomenon in inflammation, in that the cytokines are not released bacterially or virally, but by persisting metabolic derailments in the medullary space of the jawbone. If the body does not succeed in revising the metabolic disturbances in the IWH area of the jawbone within a certain period, increasing numbers of immune cells are recruited in the fatty tissue. The chronically silent inflammation of the medullary fatty tissue leads to the local development of proinflammatory signaling mediators—in particular, RANTES/CCL5. These systemically affect the organism and can result in chronic inflammatory processes or provoke further pathophysiological mechanisms.

### The problem of diagnosing FDOJ lesions by X-ray

In earlier research, we already demonstrated the nonvisibility and lack of radiographic appearance of FDOJ, which makes it difficult to obtain an accurate diagnosis by common dental radiographic means [23]. Thus, the existence of FDOJ is largely neglected today in mainstream dentistry, as is its systemic relevance. The reason for this is that conventional X-ray techniques are limited in their ability to reveal the actual extent and location of FDOJ. To aid the practitioner in diagnosing the debilitating effects of bone marrow softening inside FDOJ lesions, a computer-assisted through transalveolar ultrasound (TAU) device is available [24]. TAU precisely images and identifies cavitational porosity in the jawbone. TAU imaging proved significantly superior to radiology for the detection of microscopically confirmed FDOJ. The efficiency and reliability of TAU in the diagnosis and imaging of FDOJ has been presented in numerous publications [25]. Because of these diagnostic difficulties, jawbone disease is underdiagnosed by dentists in general and, consequently, by doctors in SYD cases. The missing coincidence between inconspicuous X-rays and the overexpression of proinflammatory signaling pathways in corresponding FDOJ areas—as shown in Figures 6, 7, and 8—lends this phenomenon importance in the discussion about “silent inflammation”.

## Conclusions

In our eyes, this is one of the first investigations to show a potential correlation between RA (of any type) and ischemic or inflammatory jawbone lesions—an association that has been speculated on for decades. Additionally, chemokine overexpression in the jawbone connected to RFT seems to be a possible danger for immune preservation, which is needed to maintain a balanced system and to prevent different types of SYD. IWH in old extraction sites and underneath RFT might also provoke immune modulation, which hinders the restoration of an already disease-modified immune system. The presence of cytokine imbalances in the jawbone leads to internal signaling through the accessory pathways via overexpressed RANTES/CCL5, which can lead to chronic pathologies such as cancer, diabetes, and cardiovascular diseases in the long term, as well as neurodegenerative or inflammatory processes. Once a chronic disease has been established, the deterioration produced by the undiscovered “silent inflammation” in the jawbone progressively creates a set of pathological conditions that worsens the overall condition and leads to further deficiencies. It is a vicious circle. This is the reason why restoring a modified system might require synergistic actions at the dental level, including surgical curettage of FDOJ or the elimination of RF with additional cleaning of the surrounding jawbone. Thus, dentists can help to not only alleviate the symptoms of acute inflammation but also put their patients on track to avoid the devastating effects of CI, which exists below the threshold of perceived pain and can smolder silently for decades. Novel therapeutic strategies that specifically target the inflammatory reaction in FDOJ could considerably contribute to the reduction of morbidity in patients. A more critical attitude inside the dental community to wound healing after dental surgery is a potential clinical implementation of the achieved results. The actual paper follows recommendations of the “EPMA White Paper” [26].

## Abbreviations

AI: acute infection
BC: breast cancer
CI: chronic inflammation
FDOJ: fatty degenerative osteonecrotic jawbone
FGF-2: fibroblast growth factor-2
HJB: healthy jawbone
IWH: insufficient wound healing
RA: rheumatoid arthritis
RF: root filling
RFT: root-filled tooth
SYD: systemic diseases
WT: wisdom tooth
WTS: wisdom tooth surgery

## References

[1] Townsend MJ, McKenzie AN (2000). Unravelling the net? Cytokines and diseases. J Cell Sci.

[2] Imbeau J (2005). Introduction to through-transmission alveolar ultrasonography (TAU) in dental medicine. Cranio.

[3] Ratner EJ, Langer B, Evins ML (1986). Alveolar cavitational osteopathosis: manifestations of an infectious process and its implication in the causation of chronic pain. J Periodontol..

[4] Mankin HJ (1992). Nontraumatic necrosis of bone (osteonecrosis). N Engl J Med..

[5] Ono K (1992). Symposium: recent advances in avascular osteonecrosis. Clin Orthop..

[6] Graff-Radford SB, Simmons M, Fox L (1988). Are bony cavities exclusively associated with atypical facial pain and trigeminal neuralgia? Proceedings of Annual Meeting.

[7] Bouquot JE, Roberts AM, Person P, Christian J (1992). NICO (neuralgia-inducing cavitational osteonecrosis): osteomyelitis in 224 jawbone samples from patients with facial neuralgias. Oral Surg Oral Med Oral Pathol..

[8] Levy JA (2009). The unexpected pleiotropic activities of RANTES. J Immunol.

[9] von Luettichau I, Nelson PJ, Pattison JM, van de Rijn M, Huie P, Warnke R (1996). RANTES chemokine expression in diseased and normal human tissues. Cytokine..

[10] Rathanaswami P, Hachicha M, Sadick M, Schall TJ, McColl SR (1993). Expression of the cytokine RANTES in human rheumatoid synovial fibroblasts. Differential regulation of RANTES genes by inflammatory cytokines. J Biol Chem..

[11] Hirano F, Kobayashi A, Hirano Y, Nomura Y, Fukawa E, Makino I (2002). Thrombin-induced expression of RANTES mRNA through protease activated receptor-1 in human synovial fibroblasts. Ann Rheum Dis.

[12] Robinson E, Keystone EC, Schall TJ, Gillett N, Fish EN (1995). Chemokine expression in rheumatoid arthritis (RA): evidence of RANTES and macrophage inflammatory protein (MIP)-1β production by synovial T cells. Clin Exp Immunol.

[13] Chicheportiche Y, Chicheportiche R, Sizing I, Thompson J, Benjamin CB, Ambrose C (2002). Proinflammatory activity of TWEAK on human dermal fibroblasts and synoviocytes: blocking and enhancing effects of anti-TWEAK monoclonal antibodies. Arthritis Res.

[14] Wang CR, Guo HR, Liu MF (2005). RANTES promoter polymorphism as a genetic risk factor for rheumatoid arthritis in the Chinese. Clin Exp Rheumatol.

[15] Soria G, Ben-Baruch A (2008). The inflammatory chemokines CCL2 and CCL5 in breast cancer. Cancer Lett..

[16] Wigler N (2002). Breast carcinoma: a report on the potential usage of the CC chemokine RANTES as a marker for a progressive disease. Isr Med Assoc J.

[17] Niwa Y, Akamatsu H, Niwa H, Sumi H, Ozaki Y, Abe A (2001). Correlation of tissue and plasma RANTES levels with disease course in patients with breast or cervical cancer. Clin Cancer Res.

[18] Huber J, Kiefer FW, Zeyda M, Ludvik B, Silberhumer GR, Prager G (2008). CC chemokine and CC chemokine receptor profiles in visceral and subcutaneous adipose tissue are altered in human obesity. J Clin Endocrinol Metab..

[19] Ye J (2009). Emerging role of adipose tissue hypoxia in obesity and insulin resistance. Int J Obes (Lond)..

[20] Kiguchi N, Kobayashi Y, Kishioka S (2012). Chemokines and cytokines in neuroinflammation leading to neuropathic pain. Curr Opin Pharmacol.

[21] Tilg H, Moschen RA (2006). Adipocytokines: mediators linking adipose tissue, inflammation and immunity. Nat Rev Immunol.

[22] Ramesh G, MacLean A, Philipp M (2013). Cytokines and chemokines at the crossroads of neuroinflammation, neurodegeneration, and neuropathic pain. Mediators Inflamm..

[23] Lechner J (2014). Validation of dental X-ray by cytokine RANTES—comparison of X-ray findings with cytokine overexpression in jawbone. Clin Cosmet Investig Dent..

[24] Bouquot JE, Margolis M, Shankland W, Imbeau J (2002). Through-transmission alveolar ultrasonography (TAU): new technology for evaluation of medullary diseases. Correlation with histopathology of 285 scanned jaw sites. Oral Surg Oral Med Oral Pathol Oral Radiol Endod..

[25] Bouquot J, Martin W, Wrobleski G (2001). Computer-based thru-transmission sonography (CTS) imaging of ischemic osteonecrosis of the jaws—a preliminary investigation of 6 cadaver jaws and 15 pain patients. Oral Surg Oral Med Oral Pathol Oral Radiol Endod..

[26] Golubnitschaja O, Costigliola V, EPMA. General Report & Recommendations in Predictive, Preventive and Personalised Medicine 2012: White Paper of the European Association for Predictive, Preventive and Personalised Medicine. EPMA J 2012, 3:14. doi:10.1186/1878-5085-3-14 10.1186/1878-5085-3-14 PMC3485619 23116135

